# The Antioxidative, Antiaging, and Hepatoprotective Effects of Alkali-Extractable Polysaccharides by* Agaricus bisporus*

**DOI:** 10.1155/2017/7298683

**Published:** 2017-08-22

**Authors:** Shangshang Li, Juan Li, Jianjun Zhang, Wenshuai Wang, Xiuxiu Wang, Huijuan Jing, Zhenzhen Ren, Zheng Gao, Xinling Song, Zhiyuan Gong, Le Jia

**Affiliations:** ^1^Institute of Agricultural Resources and Environment, Shandong Academy of Agricultural Sciences, Key Laboratory of Wastes Matrix Utilization, Ministry of Agriculture, Jinan 250100, China; ^2^College of Life Science, Shandong Agricultural University, Tai'an 271018, China; ^3^Key Laboratory of Plant Nutrition and Fertilizer, Ministry of Agriculture/Institute of Agricultural Resources and Regional Planning, Chinese Academy of Agricultural Sciences, Beijing 100081, China

## Abstract

The aim of this work was designed to investigate the antioxidant, antiaging, and hepatoprotective effects of alkali-extractable polysaccharides (AlAPS) and their three purified fractions (AlAPS-1, AlAPS-2, and AlAPS-3) from* Agaricus bisporus* in D-galactose induced aging mice. For* in vitro* antioxidant analysis, both AlAPS and its fractions exhibited moderate reducing power, Fe^2+^-chelating activities, and potent scavenging activities on hydroxyl and 1,1-diphenyl-2-picrylhydrazyl (DPPH) radicals. The* in vivo* results demonstrated that the polysaccharides, especially AlAPS-2, showed potential antiaging and hepatoprotective effects by enhancing the antioxidant status, decreasing serum hepatic enzyme activities, and improving the lipid metabolism. This study suggested that the polysaccharides extracted and purified from* A. bisporus* could be exploited as a potent dietary supplement to attenuate aging and prevent age-related diseases.

## 1. Introduction

It has been reported that the aging, as an inevitable and physiological process in living organism which can induce the gradual loss of body function and increase the probability of age-related diseases including diabetes, cognitive dysfunctions, cancer, stroke, liver failure, atherosclerosis, and Parkinson's diseases, has become a considerable challenge for health problems and disability in later life [1–5]. Recently, more and more scientists have devoted themselves on finding the mechanism; however, no accurate results are obtained. The most acceptant pathogenesis for explaining the aging process is the free radical theory initially proposed by Harman [6]. Previous literatures have demonstrated that the oxidative stress, induced by overplus of reaction oxygen species (ROS), can attack the cell membrane, nucleic acid, protein, enzymes, and other biomacromolecules by peroxidation, leading to many severe cellular and tissular damage contributing to the dysregulation of redox-sensitive signaling pathways and accelerating the progress of aging [7]. Naturally, the cells and organisms possess antioxidant enzyme systems including superoxide dismutase (SOD), catalase (CAT), and glutathione peroxidase (GSH-Px) on controlling the propagation of free radicals, limiting the formation of new free radicals, and removing the excess ROS in cells [8]. In order to reduce the oxidative damage of free radicals, a lot of synthetic antioxidants, such as butylated hydroxytoluene (BHT) and butyl hydroxy anisd (BHA), are widely used clinically and daily on preventing the formation of ROS and delaying the aging progress [9]. Nevertheless, studies have indicated that synthetic antioxidants have potential side-effects including liver damage and carcinogenesis in the long-time use [10]. Urgently, it seems necessary on exploiting natural and safe antioxidants and investigating the antioxidant and antiaging effects against aging progress.

Mushrooms, commonly used as traditional, pharmaceutical, and edible drugs/nutraceutical, have been attracting increasing attentions and become a very active domain of research in recent years owing to the physiological benefit to humans [11]. Besides, the mushrooms contain various excellent antioxidants including polyphenolic compounds, flavonoids, vitamins, polysaccharides, and carotenoids, which could prevent the ROS-induced damage and reduce the risk of chronic diseases [12]. As the most substance in the mushrooms, the polysaccharides have been isolated and confirmed to have potentially biological properties containing antioxidant, antitumor, antibacterial, immunomodulatory, antiaging, anti-inflammatory, and hepatoprotective activities [13–15], manifesting that polysaccharides have tremendous potentials for promoting human health [16].

Many literatures have demonstrated that the* Agaricus bisporus*, one of the most economically important edible mushrooms which were commonly known as the white button mushroom (WBM), has many biological activities of antitumor, antiaromatase, antimicrobial, and antioxidant activities due to the abundant nutritional components of proteins, polysaccharides, vitamins, nucleotides, and unsaturated fatty acids [17]. It is also reported that the biological activities are mainly attributed to polysaccharides [18]. However, no report about the aging effects of alkali-extractable polysaccharides (AlAPS) by* A. bisporus* against D-galactose induced aging mice has been published up till now.

In present study, the* in vivo* antioxidant activities and* in vitro* antiaging effects of AlAPS and its major fractions (AlAPS-1, AlAPS-2, and AlAPS-3) were investigated, aiming to establish the possible antiaging mechanism in the D-galactose induced aging mice. Furthermore, the molecular weight and monosaccharide compositions were also analyzed.

## 2. Materials and Methods

### 2.1. Chemicals

The fruiting body of* A. bisporus* was purchased from local commercial markets (Tai'an, China). DEAE-52 cellulose and 1,1-diphenyl-2-picrylhydrazyl (DPPH) were purchased from Sigma Chemicals Company (St. Louis, USA). The diagnostic kits for assaying activities of superoxide dismutase (SOD), glutathione peroxidase (GSH-Px), catalase (CAT), and total antioxidant activity (T-AOC), as well as contents of malondialdehyde (MDA) and lipid peroxidation (LPO), were purchased from Jiancheng Biotechnology Company (Nanjing, China). All the other chemicals and reagents were analytical grade and purchased from local chemical suppliers in China.

### 2.2. Preparation of AlAPS

The AlAPS from* A. bisporus* were performed according to a reported method with slight modifications [19]. The dried* A. bisporus* powder was extracted with proper volumes of sodium hydroxide (1 mol/L) for 6 h at 50°C (1 : 5, w/v) in a thermostatic water bath. The supernatant homogenate was precipitated with five volumes of ethanol (95%, v/v) overnight (4°C). After centrifugation (3000 rpm, 15 min), the precipitate was collected and deproteinated by the method of Miao et al. [20]. The polysaccharides content was measured by phenol-sulfuric acid method using glucose as a standard [21]. The percentage of AlAPS extraction yield was calculated according to the following formula:

(1) $$\begin{array}{c} \text{AlAPS extraction yield}\left(\;\%\;\right)=\frac{W_{i}}{W_{o}}\times 100, \end{array}$$

where *W*_*i*_ was the polysaccharide weight of extraction (g) and *W*_*o*_ represented dried sample weight (g).

### 2.3. Purification of AlAPS

The lyophilized power of AlAPS (2 g) was dissolved in distilled water (50 mL) and fractionated on the DEAE-52 cellulose anion-exchange column (1.6 cm × 20 cm) which was eluted with distilled water and then with gradient solutions of NaCl (0.1, 0.3, 0.5, and 1.0 mol/L) at a flow rate of 2 mL/tube. The major polysaccharide fractions were determined by the phenol-sulfuric acid method [21] and lyophilized for further analysis.

### 2.4. Determination of Molecular Weight

The molecular weight was analyzed using previous method with some slight modifications [22]. The polysaccharides (10 mg) were dissolved in distilled water (2 mL) and the molecular weight determination of AlAPS and its fractions were by high performance liquid chromatography (HPLC) which was operated with a HPLC system (1260, Agilent Technologies, USA) equipped with a SHODEX SB-806HQ column (8.0 mm × 300 mm) and a refractive index detector. The injection volume was 20 *µ*L. The deionized water was used as mobile phase at a flow rate of 0.5 mL/min, and the column temperature was maintained at 40°C. A series of standard dextran compounds were used to make the calibration curve.

### 2.5. Analysis of Monosaccharide Composition

The monosaccharide composition was analyzed using our previous method with some slight modifications [23]. The purified fractions were hydrolyzed with trifluoroacetic acid (TFA) at 120°C for 4 h, and the residual TFA was removed with methanol. The hydrolyzed substance was acetylated by hydroxylamine hydrochloride (12 mol/L) and sodium borohydride (dissolved in ammonium hydroxide). The reaction solution (1 *µ*L) was injected into a capillary column of Rtx-1 (30 mm × 0.25 mm × 0.25 *µ*m). The relative molar ratios of monosaccharide were calculated by the area normalization method according to the standard chromatograms of glucose (Glu), fucose (Fuc), arabinose (Ara), xylose (Xyl), ribose (Rib), galactose (Gal), mannose (Man), and rhamnose (Rha).

### 2.6. Antioxidant Activities* In Vitro*

The scavenging rate on hydroxyl radicals was measured by the method of Smironff and Cumbes with slight modifications [24]. The hydroxyl radicals were generated in the mixture of ferrous sulfate (1 mL, 9 mmol/L), salicylic acid (1 mL, 9 mmol/L), and hydrogen peroxide (1 mL, 0.03%, v/v). After addition of 1 mL polysaccharide samples (0.1–1 mg/mL), the mixture was incubated at 37°C for 30 min. The absorbance of the mixture was measured at 510 nm using water as blank. The scavenging rate on hydroxyl radicals was calculated as follows, and the EC50 value (mg/mL) was the effective concentration at which the hydroxyl radicals were scavenged by 50%.

(2) $$\begin{array}{c} \text{Scavenging rate}\left(\;\%\;\right)=\left(\;1-\frac{A_{i}}{A_{o}}\;\right)\times 100, \end{array}$$

where *A*_*i*_ was the absorbance of the polysaccharide samples and *A*_*o*_ was the absorbance of the blank.

The scavenging activity on DPPH radicals was assayed according to the method of Shimada et al. [25] with some modifications. Each sample (2 mL) was mixed with 2.0 mL DPPH (0.1 *µ*mol/L) or ethanol (95%, w/v) to a final volume of 4 mL, using distilled water (1 mL) as contrast of samples. The mixtures were shaken vigorously and left still for 30 min in the dark, and the absorbance was measured at 517 nm against ethanol as a blank. The scavenging ability was calculated as follows, and the EC50 value (mg/mL) was the effective concentration at which the DPPH radicals were scavenged by 50%.

(3) $$\begin{array}{c} \text{Scavenging ability}\left(\;\%\;\right)=1-\frac{\left(A_{i}-A_{j}\right)}{A_{o}}\times 100, \end{array}$$

where *A*_*o*_ was the absorbance of mixture containing DPPH and ethanol, *A*_*i*_ was the absorbance of mixture containing samples and DPPH, and *A*_*j*_ was the absorbance of mixture containing samples and ethanol, respectively.

The reducing power was determined according to the reported method with slight modification [22]. Each sample (1 mL) was mixed with 2.5 mL phosphate buffer (0.2 mol/L, pH 6.6), and 2.5 mL potassium hexacyanoferrate solution (1%, w/v). The mixtures were incubated 20 min at 50°C. After the reaction was cooled in flowing water, 2.5 mL trichloroacetic acid (10%, w/v), 2.5 mL distilled water, and 2.5 mL ferric chloride (0.1%, w/v) were added successively. Immediately, the absorbance of the reaction mixture was measured at 700 nm. The EC50 value (mg/mL) was the effective concentration at which the reducing power was 0.5.

The chelating effect on Fe^2+^ was measured according to the referenced method [26] with some modifications. Briefly, the reaction mixture, containing 2 mL of sample, 0.1 mL of ferrous chloride (FeCl_2_), 0.4 mL of ferrozine solution (5 mmol/L), and 5 mL of distilled water, was shaken well and incubated for 10 min at room temperature. The absorbance of the mixture was determined at 562 nm and the Fe^2+^-chelating activity was calculated according to the following formula:

(4) $$\begin{array}{c} {Fe}^{2+}\text{-chelating activity}\left(\;\%\;\right)=\frac{\left(A_{o}-A_{j}\right)}{A_{o}}\times 100, \end{array}$$

where *A*_*o*_ was the absorbance of the polysaccharide samples and *A*_*j*_ was the absorbance of the blank. The EC_50_ value (mg/mL) was the effective concentration at which the Fe^2+^-chelating activity was 50%.

### 2.7. Animal Experiments

The male Kunming mice (20 ± 2 g, purchased from Taibang Biological Products Inc., Tai'an, China) were acclimated to new conditions for 7 days with free access to food and water ad libitum at a temperature of 20–25°C under a 12 h light/dark cycle. All experiments were performed in accordance with the Regulations of Experimental Animal Administration issued by the State Committee of Science and Technology of the People's Republic of China.

The mice were randomly divided into eleven groups (five mice in each group) including three control groups of normal control (NC), model control (MC), and vitamin C positive control (PC), as well as eight dose groups of four high levels groups (400 mg/kg) and four low levels groups (200 mg/kg). Mice in low group and high group were orally fed with doses of 200 and 400 mg/kg of mice by filling the stomach (0.01 mL/g) with a syringe and D-galactose intraperitoneal injection (200 mg/kg), respectively. The mice in NC groups were treated with the same volume of physiological saline solution (0.9%, w/v). The mice in the MC groups were treated with the same volume of saline and D-galactose (200 mg/kg) intraperitoneal injection. The positive group was treated with the same volume of Vc (200 mg/kg) and D-galactose (200 mg/kg) intraperitoneal injection.

After a 30-day successive experiment, all mice were sacrificed by cervical dislocation. The blood was collected and centrifuged (10000 rpm, 4°C) for 10 min to obtain serum. The alanine aminotransferase (ALT) activity, aspartate aminotransferase (AST) activity, alkaline phosphatase (ALP) activity, albumin (ALB) levels, high density lipoprotein cholesterol (HDL-C) levels, and low density lipoprotein cholesterol (LDL-C) levels in serum were measured using automatic biochemical analyzer (ACE, USA).

The liver was surgically excised, accurately weighed, and homogenized (1 : 9, w/v) immediately in normal saline and ethyl alcohol. The homogenates were centrifuged (4000 rpm) at 4°C for 20 min, and the supernatant was obtained for analyzing the activities of SOD, GSH-Px, CAT, and T-AOC, as well as contents of MDA and LPO by the commercial reagent kits according to the instructions.

### 2.8. Statistical Analysis

All data were expressed as means ± standard deviation (SD). Statistical analyses were performed by one-way ANOVA using the SPSS statistical package (SPSS Inc. Chicago, USA). Statistical significance was considered as *P* < 0.05.

## 3. Results 

### 3.1. Isolation and Purification of AlAPS

**Figure 1:**
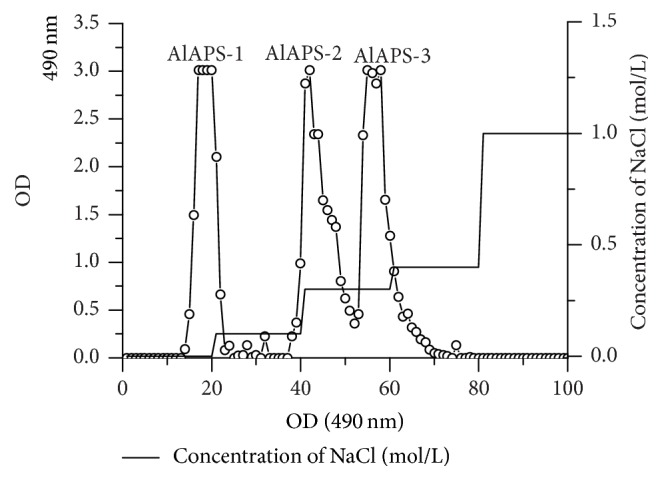
Elution profiles on DEAE-52 chromatography of AlAPS.

In present work, the yield of AlAPS was 4.31 ± 0.25%. The chromatography results of AlAPS were shown in Figure 1. Obviously, three major peaks, named as AlAPS-1, AlAPS-2, and AlAPS-3, were purified by DEAE-52 chromatography. Detailedly, the AlAPS-1 was neutral polysaccharides since it was eluted by distilled water, while the AlAPS-2 and AlAPS-3 were acidic polysaccharides since they were eluted by 0.1–0.5 mol/L NaCl solutions [27]. All the fragments were selected for the evaluation of antioxidant activities* in vitro* and* in vivo*.

### 3.2. Molecular Weight of AlAPS

According to the retention time, the molecular weight distribution of AlAPS polysaccharide was 3.47 × 10^4^ Da. The molecular weights distribution of AlAPS-1, AlAPS-2, and AlAPS-3 were 5.32 × 10^4^ Da, 2.53 × 10^4^ Da, and 7.96 × 10^4^ Da, respectively (Table 1).

### 3.3. Monosaccharide Composition

**Figure 2:**
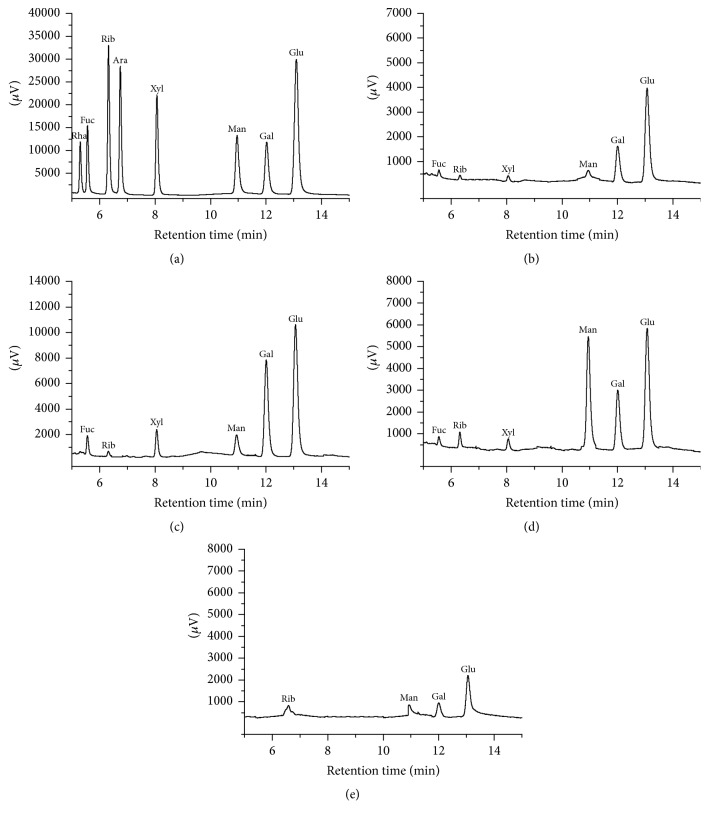
Gas chromatographs of (a) standard monosaccharides, (b) AlAPS, (c) AlAPS-1, (d) AlAPS-2, and (e) AlAPS-3.

The monosaccharide compositions of AlAPS and its three purified fractions (AlAPS-1, AlAPS-2, and AlAPS-3) were shown in Figure 2. The monosaccharides in all samples were identified by comparing the retention times with those of standards (Figure 2(a)). As shown in Figure 2(b), AlAPS contained Fuc, Rib, Xyl, Man, Gal, and Glu in a mass percentage of 12.96%, 0.58%, 1.16%, 2.2%, 28.82%, and 54.28%. As for its three fractions, AlAPS-1 was composed of Fuc (11.39%), Rib (0.61%), Xyl (5.98%), Man (7.89%), Gal (43.06%), and Glu (31.06%) with the molar ratio of 16.5 : 1 : 7.9 : 10.5 : 57 : 45.1 (Figure 2(c)), the AlAPS-2 was composed of Fuc, Rib, Xyl, Man, Gal, and Glu in a mass percentage of 6.81%, 1.98%, 2.29%, 37.39%, 22.31%, and 29.23% with the molar ratio of 3.3 : 1.1 : 1 : 16.6 : 9.9 : 14.3 (Figure 2(d)), while AlAPS-3 was composed of Rib (4.4%), Man (6.97%), Gal (22.94%), and Glu (65.69%) with a molar ratio of 1 : 1.7 : 8.5 : 24.3 (Figure 2(e)). The results indicated that the three fractions were heteropolysaccharides, Gal and Glu were the predominant monosaccharides in AlAPS, AlAPS-1, and AlAPS-3, and Man and Glu were the major monosaccharide in AlAPS-2.

### 3.4. Antioxidant Activities* In Vitro*

**Figure 3:**
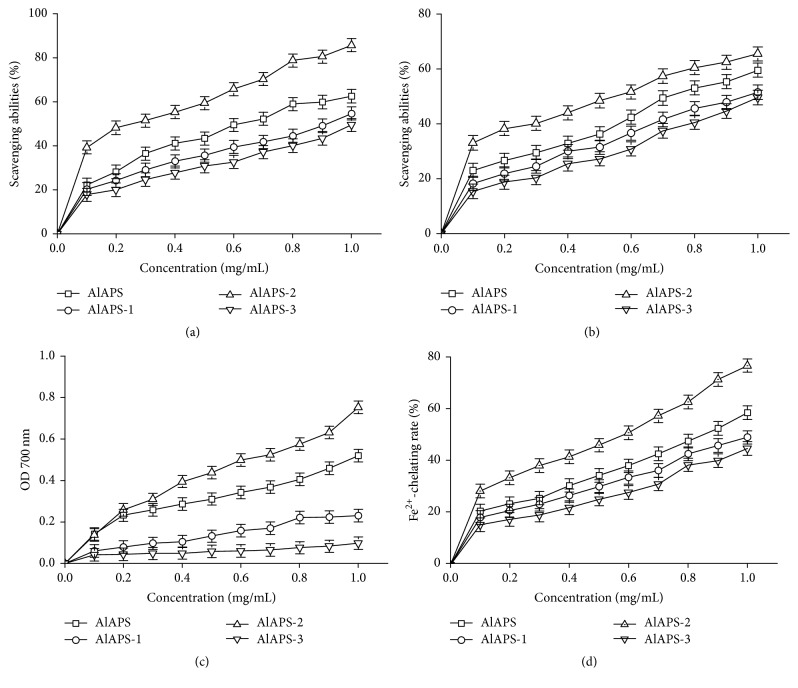
Antioxidant activities of AlAPS, AlAPS-1, AlAPS-2, and AlAPS-3* in vitro*. (a) Hydroxyl radicals, (b) DPPH radicals, (c) reducing power, and (d) Fe^2+^-chelating rate.

In this work, four parameters including reducing power, ferrous ions chelating activity, and scavenging activities on hydroxyl radicals and DPPH radicals were collected to analyze the* in vitro* antioxidant of AlAPS and its fractions, and the results were shown in Figure 3.

As shown in Figure 3(a), AlAPS, AlAPS-1, AlAPS-2 and AlAPS-3 expressed the markedly increased scavenging activities on hydroxyl radical dose-dependently. The scavenging activities of AlAPS-2 (85.78 ± 2.85%) showed considerably stronger effectivities (*P* < 0.05) than that of AlAPS (62.57 ± 3.58%), AlAPS-1 (54.67 ± 2.77%), and AlAPS-3 (49.53 ± 1.65%) at a dose of 1.0 mg/mL, respectively. Besides, the EC50 values of AlAPS, AlAPS-1, AlAPS-2, and AlAPS-3 for scavenging hydroxyl radical activity were 0.59 ± 0.23, 1.06 ± 0.03, 0.23 ± 0.07, and 1.51 ± 0.18 mg/mL, respectively.

The scavenging effects of polysaccharides on the DPPH radicals were shown in Figure 3(b). Apparently, both AlAPS and its purified fractions had obvious scavenging effects against DPPH radicals in concentration-dependent manners. Within the tested concentrations of 0–1.0 mg/mL, the scavenging activities of the AlAPS-2 (65.47 ± 1.98%) were higher than that of AlAPS, AlAPS-1, and AlAPS-3. The results were supported by EC50 values, which were found to be 0.67 ± 0.17 mg/mL for AlAPS, 1.11 ± 0.05 mg/mL for AlAPS-1, 0.305 ± 0.05 mg/mL for AlAPS-2, and 1.46 ± 0.17 mg/mL for AlAPS-3.

Figure 3(c) showed the reducing power of polysaccharides. When the polysaccharides concentrations raised from 0 to 1.0 mg/mL, the reducing power of AlAPS, AlAPS-1, AlAPS-2, and AlAPS-3 was 0.459 ± 0.05, 0.231 ± 0.11, 0.753 ± 0.08, and 0.098 ± 0.13, respectively, indicating that the AlAPS-2 had superior antioxidant activities than AlAPS, AlAPS-1, and AlAPS-3* in vitro*. The EC50 values of AlAPS-2 (1.21 ± 0.08 mg/mL) indicated that AlAPS-2 possessed stronger reducing power than AlAPS (EC_50_ = 2.14 ± 0.33 mg/mL), AlAPS-1 (EC_50_ = 3.78 ± 0.58 mg/mL), and AlAPS-3 (EC_50_ = 4.66 ± 0.67 mg/mL).

For the Fe^2+^-chelating activity, as exhibited in Figure 3(d), both AlAPS and its purified fractions showed dose-dependent manners and presented a smooth growth within the concentrations of 0–1.0 mg/mL. At the concentration of 1.0 mg/mL, the Fe^2+^-chelating rate of AlAPS, AlAPS-1, AlAPS-2, and AlAPS-3 was 58.53 ± 1.08%, 49.08 ± 2.01%, 76.68 ± 1.68%, and 44.79 ± 0.88%, respectively, indicating that AlAPS had potential antioxidant activities in chelating Fe^2+^. Additionally, the EC50 values of AlAPS, AlAPS-1, AlAPS-2, and AlAPS-3 were 1.69 ± 0.23, 2.85 ± 0.46, 0.85 ± 0.07, and 3.65 ± 0.56 mg/mL, respectively.

### 3.5. Antiaging Activities Assaying

**Figure 4:**
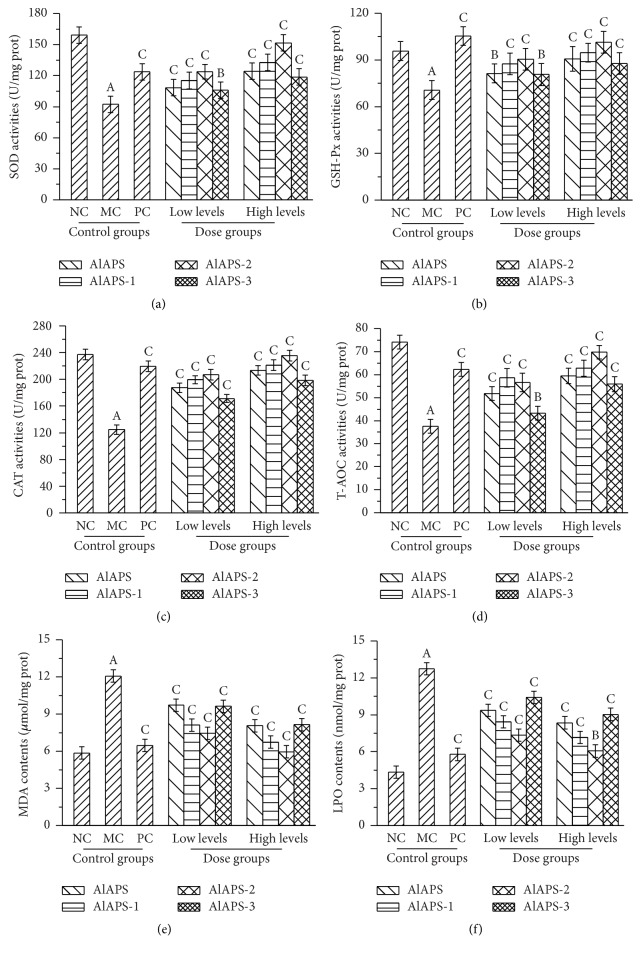
Effects of AlAPS, AlAPS-1, AlAPS-2, and AlAPS-3 on the hepatic activities of (a) SOD, (b) GSH-Px, (c) CAT, and (d) T-AOC, as well as contents of (e) MDA and (f) LPO. The values are reported as the mean ± SD of five mice per group. ^A^*P* < 0.01 compared with NC groups; ^B^*P* < 0.05 and ^C^*P* < 0.01 compared with the MC groups.

As shown in Figure 4, the hepatic activities of SOD, GSH-Px, CAT, and T-AOC of mice in the MC groups were significantly decreased when compared with that in the NC groups (*P* < 0.01), indicating that the aging models were successfully established. In this work, the hepatic activities of SOD, GSH-Px, CAT, and T-AOC were expressed dose-dependently manners by the treatment with AlAPS and its purified fractions at the tested doses of 200 and 400 mg/kg. As shown in Figure 4(a), in the high levels of AlAPS-2 groups, the SOD activities reached 151.65 ± 13.58 U/mg prot, which was higher than that of AlAPS (124.27 ± 10.54 U/mg prot), AlAPS-1 (132.87 ± 11.34 U/mg prot), and AlAPS-3 (118.72 ± 10.53 U/mg prot), respectively. As shown in Figure 4(b), significant increases in GSH-Px activities were observed after the administration of AlAPS, AlAPS-1, AlAPS-2, and AlAPS-3 at two doses of 200 mg/kg (81.34 ± 2.52, 87.48 ± 4.57, 90.41 ± 5.07, and 80.65 ± 3.58 U/mg prot) and 400 mg/kg (90.67 ± 5.73, 94.72 ± 6.47, 101.35 ± 8.24, and 87.68 ± 6.98 U/mg prot) when compared with that in the MC groups (70.65 ± 8.32 U/mg prot).  Figure 4(c) showed that the CAT activities in the mice treated with AlAPS, AlAPS-1, AlAPS-2, and AlAPS-3 reached 213.45 ± 10.54, 221.34 ± 11.02, 235.64 ± 15.14, and 198.42 ± 9.45 U/mg prot at the dosage of 400 mg/kg, which were significantly higher than that in MC groups (124.72 ± 8.44 U/mg prot, *P* < 0.01) and were almost approximate to that in the PC groups (219.43 ± 10.42 U/mg prot). In the high dose of AlAPS-2 groups, the activities of T-AOC reached the maximum of 69.73 ± 5.34 U/mg prot, which were significantly higher than those of AlAPS (59.43 ± 4.23 U/mg prot), AlAPS-1 (62.82 ± 4.97 U/mg prot), and AlAPS-3 (55.87 ± 4.07 U/mg prot) at the dose of 400 mg/kg, respectively (Figure 4(d)). Meanwhile, at a dose of 200 mg/kg, the mice in PC groups with Vc treatment also manifested significant increases of the SOD, GSH-Px, CAT, and T-AOC activities against D-galactose induced aging.

As illustrated in Figures 4(e) and 4(f), the hepatic contents of MDA and LPO were significantly increased (*P* < 0.01) in the D-galactose induced aging mice when compared with that in the NC groups. Interestingly, AlAPS and its fractions significantly suppressed the contents of MDA and LPO dose-dependently. As shown in Figure 4(e), the MDA contents of AlAPS-2 in liver reduced to 5.94 ± 0.11 *µ*mol/mg, which was about 52.63% lower than that of MC groups when compared with that in the MC groups (12.07 ± 0.23 *µ*mol/mg), and almost approximate to that in the NC groups (5.85 ± 0.15 *µ*mol/mg), respectively. As shown in Figure 4(f), the LPO contents of AlAPS-2 at the dose of 400 mg/kg reached 6.06 ± 0.15 nmol/mg prot (*P* < 0.01), lower than those in other dose groups, respectively. The positive group also demonstrated the significant effect compared with MC group (*P* < 0.01).

In this work, these data indicated that AlAPS and its purified fractions had potential antiaging effects by improving SOD, GSH-Px, CAT, and T-AOC activities, as well as reducing MDA and LPO contents in the liver tissue against D-galactose induced aging.

### 3.6. Biochemical Assays in Serum

**Figure 5:**
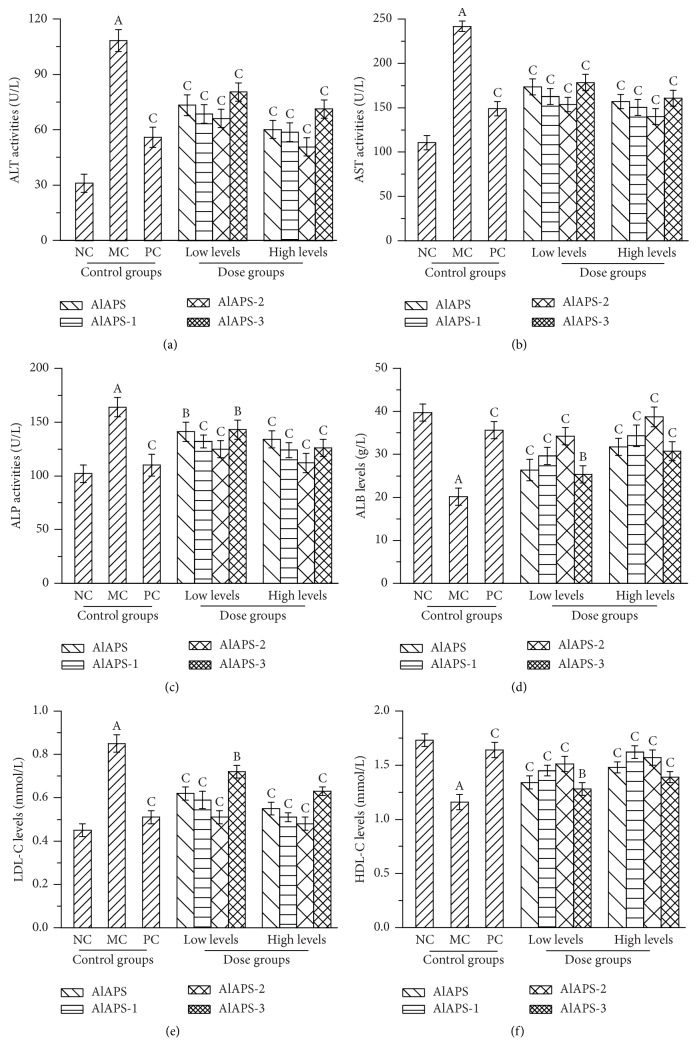
Effects of AlAPS, AlAPS-1, AlAPS-2, and AlAPS-3 on (a) ALT activities, (b) AST activities, (c) ALP activities, (d) ALB levels, (e) LDL-C levels, and (f) HDL-C levels in D-galactose induced aging mice. The values are reported as the mean ± SD of five mice per group. ^A^*P* < 0.01 compared with NC groups; ^B^*P* < 0.05 and ^C^*P* < 0.01 compared with the MC groups.

Several enzymes in serum such as ALT, AST, and ALP were usually used as biochemical markers of liver damage. As displayed in Figure 5, when compared with that in the NC groups, the mice in MC groups showed serious liver damage as evidenced by significant increases in the activities of ALT, AST, and ALP, as well as levels of ALB (with all *P* < 0.01). As shown in Figure 5(a), The ALT activities reached 50.9 ± 3.5 U/L (*P* < 0.01) in AlAPS-2 group at the dosage of 400 mg/kg, which were lower than that in MC groups (108.5 ± 5.4 U/L, *P* < 0.01). As for AST and ALP, the minimum activities reached 140.1 ± 7.5 U/L and 112.1 ± 8.3 U/L, which were significantly lower than that in MC groups (241.6 ± 12.5 U/L and 164.3 ± 9.3, Figures 5(b) and 5(c), both *P* < 0.01), respectively.  Figure 5(d) showed that the levels of ALB in the D-galactose induced aging mice were significantly higher than those in the MC groups (20.15 ± 0.83 g/L, *P* < 0.01). Furthermore, the AlAPS-2 (38.69 ± 2.05 g/L) showed superior effects when compared to AlAPS (31.72 ± 1.63 g/L), AlAPS-1 (34.31 ± 1.34 g/L), and AlAPS-3 (30.78 ± 0.96 g/L) at a dose of 400 mg/kg, respectively. Meanwhile, the Vc at a dose of 200 mg/kg demonstrated significant antiaging effects against D-galactose. The results indicated that supplementation with AlAPS and its fractions had potential protection on aging liver by depressing the activities of ALT, AST, and ALP, as well as levels of ALB. As Figures 5(e) and 5(f) displayed, significant reductions of HDL-C levels and significant elevations of LDL-C levels were observed in MC groups as compared to the NC groups (*P* < 0.01). The LDL-C levels in group of AlAPS-2 at 400 mg/kg were 0.48 ± 0.03 mmol/L, which was lower than that in MC groups (Figure 5(e), *P* < 0.01). As shown in Figure 5(f), in the dosage groups of 400 mg/kg treated with AlAPS-1, the HDL-C levels were 1.62 ± 0.07 mmol/L, which was more approximate to that in the PC group. These results signified that AlAPS and its purified fractions had potential effects in amelioration of liver damage.

## 4. Discussion

In recent years, aqueous extraction of polysaccharides from* A. bisporus* fruiting bodies, which were reported to possess a significantly antioxidant activity, have been receiving more and more attentions [18]. Nevertheless, few studies have focused on the alkali-extractable polysaccharides from* A. bisporus* fruiting bodies. The present study exhibited that AlAPS were considered as representative heteropolysaccharides due to the elution purified by DEAE-52 cellulose column and gas chromatograph.

A review of recent literatures had revealed that the aging-related hepatoprotective activities of natural substances were related to antioxidant and free radical scavenging activities [28, 29]. The overproduction of ROS, including superoxide anion radical, hydroxyl radical, hydrogen peroxide, reactive oxygen, DPPH radicals, and nitrogen species, could result in oxidative stress, leading to the chemical modification of key cellular components, DNA instability, the membrane integrity, and decline in enzymatic activities [30]. Previous literatures had reported that the hydroxyl radicals, as the main reactive oxygen species in biological tissues, can react with most bimolecular compounds including amino acids, lipids, and DNA, causing the general aging processes of tissue damage [10]. Experimentally, the DPPH-based assay system was a rapid and efficient method for measuring the free radical scavenging activities of natural compounds; the possible mechanism may be that DPPH radicals could accept an electron/hydrogen to become a stable diamagnetic molecule, with the reduced absorbance [31]. The reducing capacity of a compound may serve as a significant indicator of its potential antioxidant activity, contributing to the potential breaking effects on the free radical chain [32, 33]. Furthermore, the nonenzymatic metal of Fe^2+^ chelating may be considered as an important antioxidant property by retarding the metal-catalyzed oxidation [34]. In present work, AlAPS-2 showed stronger scavenging abilities than AlAPS and other fractions on hydroxyl and DPPH radicals. Similar results were also observed on Fe^2+^-chelating abilities and reducing power. Previous research had reported that the molecular weight of polysaccharides was an important parameter, which could influence the antioxidant activity [35]. The AlAPS and AlAPS-2 with the lower molecular weights showed higher antioxidant activities, indicating that a relatively low molecular weight may enhance the antioxidant activity. Our results were also confirmed by Chen et al. [35]. Additionally, several literatures have reported that the antioxidant properties of polysaccharides were mainly associated with their monosaccharide compositions [36]. As shown in monosaccharide analysis, Gal and Glu were the predominant monosaccharides in AlAPS, AlAPS-1, and AlAPS-3, Man and Glu were the major monosaccharides in AlAPS-2, which is in agreement with the* in vivo* antioxidant activities, and Man was monitored in AcAPS-2, indicating that Man may play important roles in maintaining the aging effects.

Clinically, the activities of ALT, AST, and ALP in serum had been used as biochemical markers for liver damage, which could be leached out of hepatocytes into blood circulation when the permeability of cell membrane is damaged. When liver damage occurred, the enzymes activities were markedly heightened in serum [37]. Our results demonstrated that D-galactose induced aging showed serious liver damage as evidenced by significant elevations of the ALT, AST, ALP, and ALB. And the AlAPS and its fractions had hepatoprotective effects by stabilizing the plasma membrane as well as repairing the hepatic tissue damage. Increased HDL-C levels and decreased LDL-C levels were a curial advantage in improving hyperlipemia in oxidative stress mice [38]. These results suggested that both AlAPS and its purified fractions had potential inhibition against aging-induced liver injury.

According to the previous literatures, the D-galactose-mediated aging-toxicity at low dose has been widely used in applying for inducing aging properties by engendering lipid peroxidation and depletion of the antioxidant enzymes and eventually aging-related organic diseases of liver damage [39, 40]. Antioxidant enzymes consisting of SOD, GSH-Px, and CAT, regarded as the first line of the antioxidant defense against ROS generated during the oxidative stress* in vivo*, were considered to be the primary defense that prevents biological macromolecules from potentially injurious oxidizing agents [1, 39]. Briefly, the SOD showed potential antiaging effects by decomposing the superoxide radical to hydrogen peroxide [41]. Meanwhile, the GSH-Px, regarded as a selenium-containing enzyme against ROS, could directly react with ROS on preventing the formation of H_2_O_2_-induced hydroxyl radicals [1]. As a member of enzyme in the peroxisomes family, the CAT could facilitate the metabolism of hydrogen peroxide, resulting in the less-reactive substances of gaseous oxygen and water molecules [8]. Besides the enzymatic antioxidant systems, the nonenzymatic antioxidant systems (reflected by T-AOC activities presently) showed potential effects against the various ROS formation, increasing the susceptibility to oxidative damage [42, 43]. In addition, both MDA and LPO, considered as the end products of lipid peroxidation, could be deemed to the hallmarks of oxidative stress causing aging and its applications (hepatic injuries) [21, 44, 45]. In this work, the D-galactose injection caused the serious oxidative stress reflected by the significant decreases of SOD, GSH-Px, CAT, and T-AOC activities, as well as significant increases of MDA and LPO contents in aging mice. Interestingly, the treatment with AlAPS and its fractions (AlAPS-1, AlAPS-2, and AlAPS-3) remarkably enhanced the antioxidant enzyme activities and reduced the lipid peroxidation contents, indicating that the polysaccharides extracted and purified form* A. bisporus* could remit and improve the aging properties either by scavenging excessive free radicals or by promoting antioxidant enzyme activities.

## 5. Conclusions 

In conclusion, both AlAPS and its purified fractions of AlAPS-1, AlAPS-2, and AlAPS-3 from* A. bisporus* have shown to be an excellent source of antioxidant, antiaging, and hepatoprotective polysaccharides against D-galactose induced aging in mice, which would be exploited as a potentially natural and functional food or a novel nutraceutical for the prevention and alleviation of aging and its complications.

## Figures and Tables

**Table 1:** Molecular weights of AlAPS.

	Mw, ×10^4^ Da
AlAPS	3.47
AlAPS-1	5.32
AlAPS-2	2.53
AlAPS-3	7.96

## Abbreviations

AlAPS: Alkali-extractable polysaccharides
ALP: Alkaline phosphatase
ALT: Alanine aminotransferase
Ara: Arabinose
AST: Aspartate aminotransferase
BUN: Blood urea nitrogen
CAT: Catalase
GC: Gas chromatography
CER: Ceruloplasmin
D-gal: D-Galactose
DPPH: 1,1-Diphenyl-2-picrylhydrazyl
Fuc: Fucose
Glu: Glucose
GSH-Px: GSH peroxide
HDL-C: High density lipoprotein cholesterol
H_2_O_2_: Hydrogen peroxide
LDL-C: Low density lipoprotein cholesterol
LPO: Lipid peroxidation
Man: Mannose
MC: Model control
MDA: Malondialdehyde
NC: Normal control
PC: Positive control
prot: Protein
Rib: Ribose
Rha: Rhamnose
ROS: Reactive oxygen species
SOD: Superoxide dismutase
T-AOC: Total antioxidant capacity
Xyl: Xylose.
